# PVDF-Based Piezoelectric Pressure Sensor for Wearable Throat-Motion Monitoring

**DOI:** 10.3390/polym18212766

**Published:** 2026-11-06

**Authors:** Delang Lin, Wei Zhong, Yayi Chen, Ming Li

**Affiliations:** 1 The School of Integrated Circuits, Guangdong University of Technology, Guangzhou 510006, China; eelindl@ust.hk (D.L.); zwnice@163.com (W.Z.); chenyy@gdut.edu.cn (Y.C.); 2 The School of Marine Science and Engineering, South China University of Technology, Guangzhou 510640, China

**Keywords:** Polyvinylidene fluoride, thin-film transistor, pressure sensing, wearable electronics, throat-motion monitoring

## Abstract

Thin piezoelectric polymers provide a compliant route for converting transient biomechanical deformation into electrical signals, but wearable sensing also depends on how these high impedance signals are coupled to the electronic readout. This work presents a hybrid sensing platform that combines a piezoelectric film, an InSnZnO (ITZO) dual-gate thin-film transistor (DGTFT), and analog signal conditioning for dynamic force measurement and anterior neck motion sensing. The polymer film contains identifiable electroactive crystalline features and generates repeatable bipolar responses under cyclic loading. The coupled device exhibits gate-dependent drain current responses, while the complete readout provides a force-to-voltage sensitivity of 4.0 V/N over 0.5–1 N. Under continuous 0.5 N cyclic loading for 200 s, the output shows a 3.12% coefficient of variation and a 102.27% final-to-initial response ratio. Neck-mounted measurements further resolve different temporal signatures associated with coughing, swallowing, and pronunciation of “hello”. The results demonstrate a compact polymer-transistor sensing architecture that preserves dynamic mechanical information from controlled loading to wearable biomechanical acquisition.

## 1. Introduction

Wearable mechanical sensing translates deformation, pressure, vibration, and displacement at the body surface into measurable electrical signals. Unlike conventional rigid transducers, skin-mounted devices must operate on curved and continuously moving interfaces while retaining information carried by transient events. This requirement is particularly relevant to the anterior neck, where respiration, phonation, coughing, and swallowing generate mechanical activity over different amplitudes and timescales. The resulting signals contain information in their temporal structure, including transient duration, polarity changes, peak distribution, repetition, and waveform envelope [1,2].

Piezoelectric polymers offer a direct route for detecting these dynamic mechanical events because deformation changes the polarization state of the material and generates an electrical response without requiring a resistive or capacitive deformation mechanism. Polyvinylidene fluoride (PVDF) is particularly attractive for wearable implementations owing to its low density, mechanical flexibility, chemical stability, and compatibility with thin-film form factors [3,4]. Its electromechanical behavior arises from the semi-crystalline polymer structure, in which molecular chain conformation, crystalline phase distribution, dipole orientation, and the interaction between crystalline and amorphous regions determine the polarization response under deformation [3].

Research on PVDF sensing has developed along several complementary routes. Material-oriented studies regulate electroactive phases through stretching, poling, thermal processing, nanofillers, and interfacial engineering [3,5]. Device-oriented studies employ porous films, electrospun fibers, composite layers, textiles, and multilayer structures to redistribute stress and improve mechanical conformity [4,5]. These strategies have enabled applications ranging from tactile loading and joint motion to arterial pulse, respiration, vocal vibration, and swallowing [6,7]. The diversity of these designs also shows that the measured response is determined by the complete mechanical–electrical pathway [8].

Moreover, a second design problem emerges when a piezoelectric film is connected to electronic readout. Piezoelectric polymers behave as high-impedance dynamic sources, so the electrical quantity available to the acquisition system depends on the interface between the transducer and the following circuit. Oxide thin-film transistors (TFTs) have consequently been introduced into piezoelectric sensing architectures as electronically addressable interfaces, with reported systems using piezoelectric potentials to produce transistor-current responses and active readout functions [9,10,11].

The anterior neck provides a demanding test case for such an interface because its mechanical signals are heterogeneous. Coughing contains rapid transient motion, swallowing involves coordinated movement of multiple cervical structures, and phonation produces repetitive vibration transmitted through laryngeal tissues. Therefore, recent neck-mounted systems have moved beyond simple event detection toward waveform characterization using temporal, spectral, and time–frequency features [2,12]. For a piezoelectric platform, preserving these differences requires both a mechanically compliant transducer and an electrical acquisition chain that can retain the dynamic structure of the original event.

These developments motivate a system-level view of piezoelectric sensing in which the polymer, transistor interface, and analog readout are treated as successive functional elements. PVDF provides a reproducible and readily available mechanical transducer, while transistor coupling introduces an electronic signal domain between the raw piezoelectric response and the final acquisition voltage. Characterizing these domains separately offers a direct way to relate material response, electronic conversion, quantitative force measurement, and wearable signal acquisition.

A hybrid PVDF-ITZO sensing platform is developed in this work to investigate this multistage mechanical-to-electrical pathway. A PVDF film serves as the dynamic mechanical transducer, an ITZO TFT provides the transistor-level electrical interface, and an analog circuit generates the final voltage output used for quantitative sensing. The study combines material characterization, transducer level measurement, transistor domain acquisition, force calibration, repeated operation evaluation, and anterior-neck sensing within the same device framework. This organization enables the progression from polymer electromechanics to system-level biomechanical acquisition to be examined within a unified experimental platform.

## 2. Materials and Methods

The dual-gate thin-film transistor (DGTFT) was fabricated on a polyimide substrate with an Al bottom gate, Al_2_O_3_ bottom-gate dielectric, ITZO active layer, ITO source/drain electrodes, ZrO_2_ top-gate dielectric, and Al top gate. The channel width and length were both 300 μm. The ITZO layer was deposited by co-sputtering ZnO and ITO targets at 0.5 Pa using target powers of 130 and 100 W, respectively. The Ar/O_2_ flow rates were 10 and 6 sccm, respectively. The device was annealed in air at 300 °C for 3 h.

A commercial pre-poled PVDF piezoelectric film from the PVDF-Piezo-1 product series (PolyK Technologies, LLC, State College, PA, USA), with a nominal thickness of 28 μm, was used. The film was manufactured by melt extrusion, followed by uniaxial orientation and corona poling. The piezoelectric coefficient magnitudes are |d_31_| = 25 pC/N, |d_33_| = 20 pC/N, and |d_32_| < 5 pC/N. The relative dielectric constant is approximately 13, and Young’s modulus along the orientation direction exceeds 2.0 GPa at 20 °C. Aluminum electrodes were deposited on both surfaces of the PVDF film by thermal evaporation through a metal shadow mask. Each electrode had a nominal thickness of approximately 300 nm and dimensions of 10 mm × 10 mm. For electrical coupling, one Al electrode of the PVDF film was connected to the top gate of the ITZO DGTFT using low-temperature conductive silver paste, while the opposite Al electrode was connected to the common electrical reference ground. The bottom gate was independently biased to control the transistor operating condition.

X-ray diffraction (XRD) measurements were performed using a D8 Advance diffractometer (BrukerAXS SE, Karlsruhe, Germany) with Cu Kα radiation (Kα_1_ = 1.54056 Å and Kα_2_ = 1.54433 Å). The diffraction pattern was collected over a 2*θ* range of 3–60° with a step size of 0.01° and a scan rate of 2° min^−1^. The diffraction features around 18.4° and 20–21° were examined because they correspond to characteristic reflections reported for PVDF crystalline phases. The observed diffraction angles are related to the corresponding interplanar spacings through Bragg’s law.

Atomic force microscopy (AFM) was performed on the pristine PVDF film before electrode deposition using a Jupiter XR microscope (Oxford Instruments, Asylum Research, Santa Barbara, CA, USA) equipped with a TITAN300 silicon probe. The probe had a nominal resonance frequency of 300 kHz, a spring constant of 26 N/m, and a tip radius of approximately 7 nm. Cantilever deflection was monitored using an optical beam-deflection system, while closed-loop positioning was supported by linear variable differential transformer (LVDT) sensors. Surface topography was acquired in AC tapping mode over a 10 × 10 μm^2^ scanning area at approximately 1.00 Hz with a resolution of 256 × 256 pixels. Surface roughness parameters were calculated from the acquired height data.

**Figure 1:**
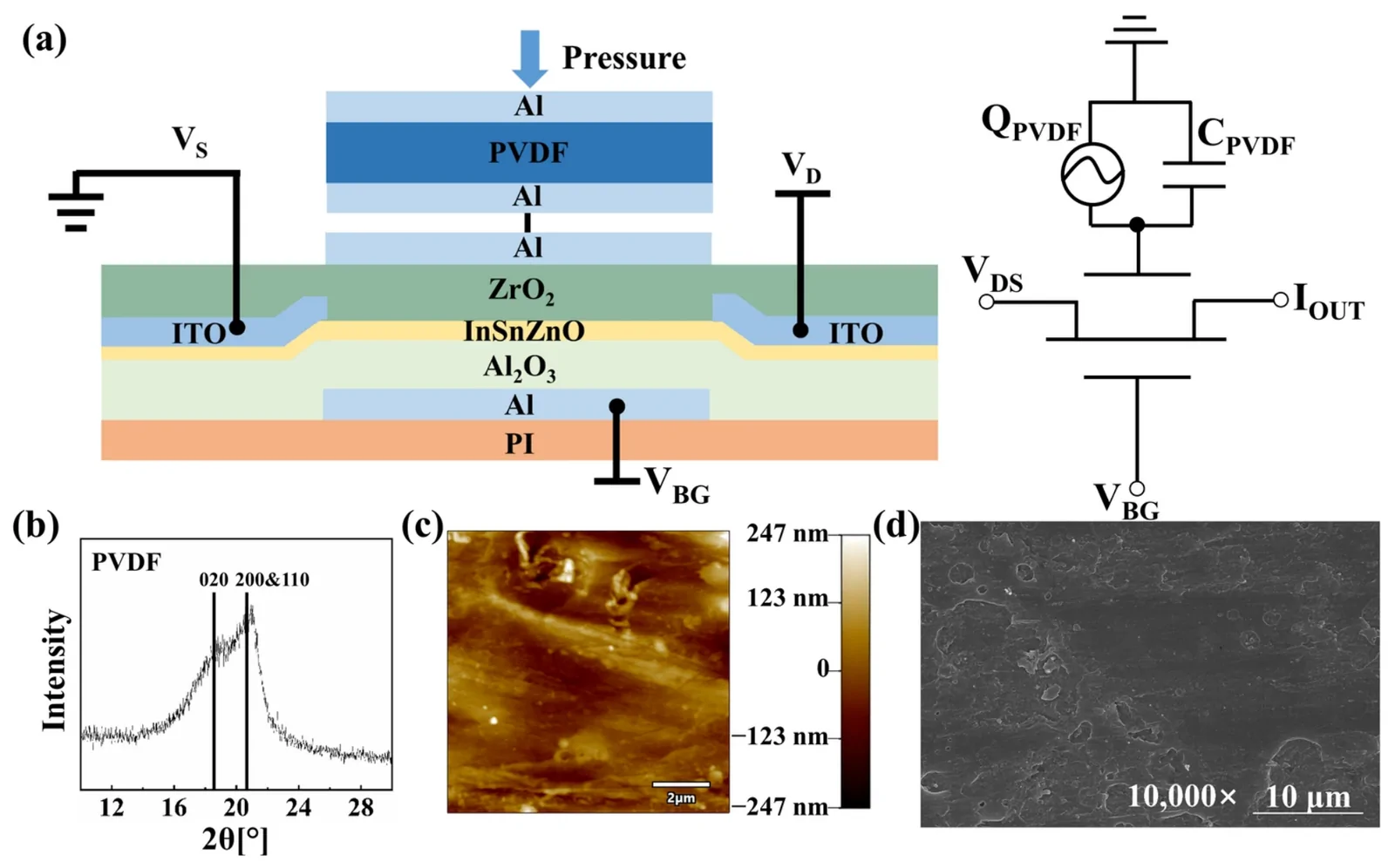
(**a**) Schematic of the integrated PVDF-ITZO sensing platform. (**b**) XRD pattern of the PVDF film. (**c**) AFM surface morphology and (**d**) SEM image of the pristine PVDF film.

Scanning electron microscopy (SEM) of the pristine PVDF surface before Al deposition used an Apreo 2 C microscope (FEI Company, part of Thermo Fisher Scientific, Hillsboro, OR, USA) at 10 kV, 50 pA, and a working distance of 10.8 mm with a T2 detector. A representative image was acquired at 10,000× (Figure 1d).

The electrical characteristics of the ITZO dual-gate TFT were evaluated through transfer and bottom-gate leakage current measurements. During these measurements, the TFT top gate remained electrically connected to one Al electrode of the PVDF film, while the opposite Al electrode was connected to the circuit reference ground. The bottom-gate voltage (*V*_BG_) was swept from −20 to 20 V, with the drain-source voltage (*V*_DS_) fixed at either 0.1 or 10.1 V. The standalone PVDF film was tested under repeated normal loading of 1 N, and its voltage response was recorded as *V*_PVDF(t)_. For the coupled PVDF-TFT measurements, drain-current transients were recorded at *V*_DS_ = 0.5 V with *V*_BG_ = 0, 1, 2, and 3 V. Force calibration, short-term repeatability testing, and neck-motion measurements were conducted at fixed biases of *V*_DS_ = 0.5 V and *V*_BG_ = 0 V.

The complete device was subsequently connected to the analog readout circuit, and the final signal was measured at *V*_OUT_. The analog readout circuit consists of a transimpedance amplifier (*U*_1_) and a non-inverting voltage amplifier (*U*_2_), both employing TLC2201ACD operational amplifiers (Texas Instruments) supplied with ±5 V. The first stage provides a nominal low-frequency transimpedance of 10 MΩ, with a calculated −3 dB cutoff frequency of approximately 72.3 Hz, determined by the R_2_-C_1_ feedback network. The second stage provides a voltage gain of 4 V/V. The R_6_-C_2_ output RC low-pass filter has a calculated −3 dB cutoff frequency of approximately 1.04 kHz. The conditioned output voltage *V*_OUT_ was acquired using a PicoScope 2204A USB oscilloscope (Pico Technology) at a sampling rate of 1 kS/s, without additional digital filtering.

Mechanical loading was applied using a manual force gauge and a loading head with a contact area of 10 mm^2^. Force levels of 0.5, 0.75, and 1 N were investigated under the same electrical acquisition configuration, with the loading cadence guided by a metronome set to 2 Hz. Twenty complete loading response events were recorded and analyzed at each force level. For each event, the positive peak output voltage was defined as the maximum recorded *V*_OUT_ within the corresponding loading response window. The mean and sample standard deviation of the 20 positive peak voltages were calculated for each force level, and the three mean values were used for linear calibration of the complete sensing system.

The force-to-voltage sensitivity was defined as

(1) $$S_{F}=\frac{∆V_{OUT}}{∆F},\;$$

The calibration data were fitted using

(2) $$V_{OUT}=S_{F}F+V_{0},\;$$

where *V*_0_ is the fitted intercept. The coefficient of determination *R*^2^ was obtained from the linear regression.

For short-term repeatability measurements, a cyclic load of 0.5 N was applied at a nominal frequency of 1 Hz for 200 s, corresponding to approximately 200 loading cycles. Each complete loading release response was treated as an individual analysis window, and the maximum recorded output voltage within the corresponding window was extracted as the positive peak voltage. Response retention was calculated as

(3) $$R_{ret}=\frac{{\bar{V}}_{final}}{{\bar{V}}_{initial}}\times 100\%,\;$$

where ${\bar{V}}_{initial}$ and ${\bar{V}}_{final}$ represent the mean positive peak voltages of the first and last 10 complete response events, respectively.

The event-to-event variation in the positive peak voltage sequence over the complete 200 s measurement was quantified using the coefficient of variation:

(4) $$CV=\frac{\sigma_{V}}{\bar{V}}\times 100\%,\;$$

where $\sigma_{V}$ and $\bar{V}$ denote the sample standard deviation and arithmetic mean of the extracted positive peak voltages, respectively.

For neck-motion measurements, the PVDF sensing element was encapsulated with a polyimide (PI) insulating layer and attached to the anterior neck near the laryngeal prominence using medical adhesive tape. The PI layer prevented direct contact between the skin and the Al electrodes and covered the exposed conductive silver paste contacts to provide electrical insulation. One Al electrode of the PVDF film was connected to the top gate of the ITZO dual-gate TFT, while the opposite electrode was connected to the common electrical ground. The connecting leads were secured using medical adhesive tape to minimize unintended cable movement during measurement. Coughing, swallowing, and pronunciation of ‘hello’ were repeatedly performed by the same volunteer.

The recorded signals were evaluated in the time domain. Waveform comparison included amplitude, temporal extent, peak distribution, polarity sequence, and overall morphology. Each complete throat-motion action was treated as an individual event for quantitative analysis. The recorded voltage signals were represented at a temporal resolution of 1 ms. Event centers were identified offline from the recorded voltage envelope, while the peak-to-peak voltage of each event was determined directly from the original sampled waveform within the corresponding event window. The peak-to-peak voltage was defined as

(5) $$V_{pp}=V_{max}-V_{min},\;$$

where $V_{max}$ and $V_{min}$ are the maximum and minimum recorded voltages within the corresponding event window, respectively. The repeated event values were summarized using the arithmetic mean, sample standard deviation (SD), and coefficient of variation (CV = SD/mean × 100%).

## 3. Results and Discussion

The integrated sensing platform combines an Al/PVDF/Al piezoelectric element with an ITZO DGTFT, as illustrated in Figure 1a. The PVDF element forms the mechanically active part of the device, while the transistor and subsequent electrical circuitry provide the electronic pathway for signal acquisition. The ITZO device consists of an Al bottom gate, Al_2_O_3_ bottom-gate dielectric, ITZO active layer, ITO source/drain electrodes, ZrO_2_ top-gate dielectric, and Al top gate on a PI substrate. In the equivalent representation, the PVDF element is described by the piezoelectric source *Q*_PVDF_ together with its capacitance *C*_PVDF_, providing a compact description of the electromechanical sensing element.

The XRD pattern in Figure 1b presents the characteristic diffraction region of PVDF from 10° to 30°, selected from the full 2*θ* scan of 3–60° to highlight the reflections near 18.4° and 20.6°. A relatively weak diffraction feature appears near 2*θ* = 18.4°, corresponding to the (020) reflection of the nonpolar α phase, while a stronger feature near 2*θ* = 20.6° is consistent with the overlapping (110)/(200) reflections commonly associated with the electroactive β phase [13,14]. These diffraction features indicate the semi-crystalline structure of the PVDF film, with contributions from the α phase and electroactive crystalline regions. The pronounced diffraction feature around 20.6° is particularly relevant to piezoelectric sensing because the polar molecular chain arrangement associated with the β phase provides a structural basis for polarization changes under mechanical deformation [13]. Nevertheless, because the characteristic diffraction reflections of different crystalline phases may overlap, possible contributions from the γ phase in the 20–21° region cannot be excluded. Therefore, the crystalline-phase characteristics are just discussed qualitatively.

The AFM height map of the pristine PVDF film in Figure 1c, acquired over a 10 × 10 μm^2^ area, reveals elongated surface textures, local protrusions, and distributed height variations. The arithmetic mean height (S_a_) is 17.418 nm, while the root mean square height (S_q_) is 22.176 nm. These measurements characterize the local surface morphology of the PVDF film before electrode deposition.

The SEM image in Figure 1d shows a relatively continuous PVDF surface containing fine, partially oriented striations and localized morphological irregularities. These features are qualitatively consistent with the elongated surface textures and local height variations observed in the AFM measurements. Together, AFM and SEM provide complementary information on the pristine film surface used for device fabrication.

**Figure 2:**
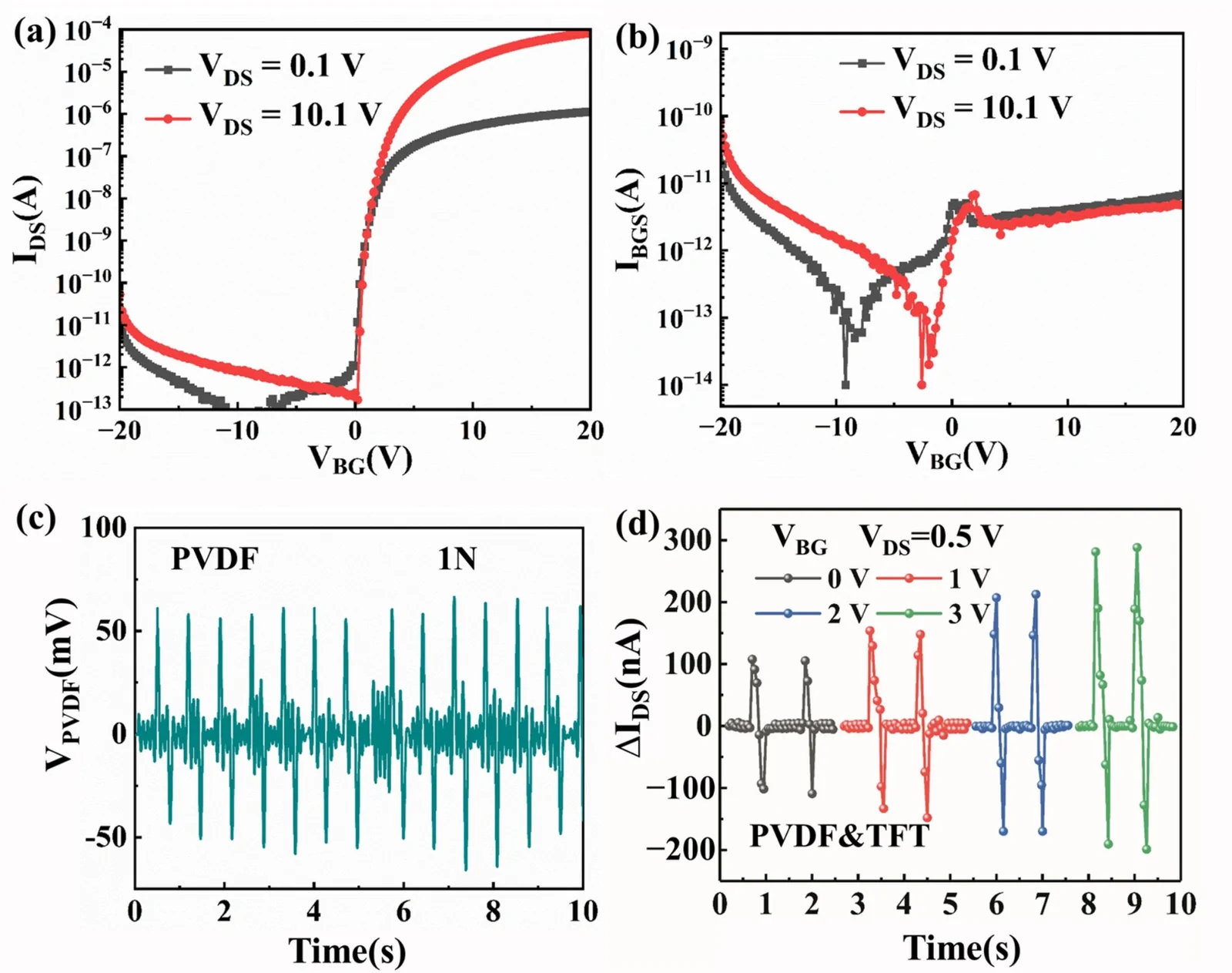
(**a**) Transfer characteristics and (**b**) gate-leakage current of the ITZO dual-gate TFT. (**c**) Voltage response of the PVDF film under repeated 1 N loading. (**d**) Drain-current responses of the PVDF-ITZO device at different gate voltages under repeated 1 N loading.

The structural and morphological characteristics of PVDF influence different aspects of its electromechanical response. From a structural perspective, the polar molecular chain arrangements associated with electroactive crystalline regions, together with the prior poling of the film, provide the basis for a net polarization response to mechanical deformation. Because PVDF is a semi-crystalline polymer, the crystalline regions are embedded within an amorphous matrix, and externally applied stress is transferred through both components. Deformation of this coupled structure changes the polarization state of the film and consequently the charge induced on its electrodes. This interpretation is consistent with the electrical response measured in Figure 2c, where the PVDF film generates repeated bipolar voltage transients with amplitudes on the order of tens of millivolts under cyclic loading of 1 N. The positive and negative voltage excursions reflect dynamic charge generation and redistribution during mechanical deformation and release.

From an interfacial perspective, the surface morphology observed by AFM and SEM is relevant to the formation of the Al electrodes and the mechanical boundary conditions during loading. Local surface protrusions and height variations may affect the uniformity of electrode contact and the spatial distribution of stress transmitted into the PVDF layer, thereby influencing the local electromechanical response. Thus, the crystalline structure provides the physical basis for piezoelectric charge generation, while the surface morphology is relevant to the mechanical coupling and electrode interface through which that response is obtained.

The transfer characteristics and gate leakage currents of the ITZO DGTFT were measured over a bottom-gate voltage range of −20 to 20 V at drain-source biases of 0.1 and 10.1 V, as shown in Figure 2a,b. The transfer curves exhibit a pronounced turn-on transition near *V*_BG_ = 0 V and a low off-state drain current of approximately 1 pA. The corresponding gate-leakage measurements indicate effective gate insulation over the investigated bias range. These characteristics support the selection of *V*_BG_ = 0 V as the operating condition for piezoelectrically induced drain-current modulation with relatively low quiescent channel conduction.

The electromechanical behavior of the PVDF element is directly reflected in its response to repeated 1 N loading. As shown in Figure 2c, consecutive mechanical events generate well-resolved bipolar voltage transients throughout the recording period. Positive peaks reach the tens of millivolts level and are accompanied by negative excursions during the cyclic response. The repeated appearance of opposite-polarity peaks is characteristic of dynamic piezoelectric conversion, in which changes in mechanical deformation produce transient polarization and charge redistribution within the PVDF element [3,8].

The dynamic behavior can be described by the direct piezoelectric relation

(6) D=dT+εE,

where D denotes electric displacement, T is the applied mechanical stress, d is the piezoelectric coefficient, E is the electric field, and ε is the permittivity. From an electrical viewpoint, a piezoelectric film can be represented as a mechanically driven charge source associated with its intrinsic capacitance [8]. Consequently, cyclic deformation naturally gives rise to transient electrical signals whose polarity follows the direction of the polarization change [3,8]. This interpretation connects the bipolar waveform in Figure 2c with the intrinsic dynamic electromechanical behavior of PVDF [3].

The dual-gate configuration provides separate electrical interfaces for piezoelectric signal coupling and transistor bias control. The voltage generated by the PVDF film is applied to the top gate, where it modulates the electrostatic potential of the ITZO channel and produces transient changes in the drain current. The bottom-gate voltage independently adjusts the channel operating condition. Under repeated 1 N loading at *V*_DS_ = 0.5 V, the magnitude of the transient drain current response increases progressively as *V*_BG_ rises from 0 to 3 V (Figure 2d). This trend is consistent with the enhanced channel conduction at more positive bottom-gate biases observed in Figure 2a. Increased channel conduction is also associated with a higher quiescent drain current and greater static TFT power consumption at a fixed drain-source voltage. Therefore, the bottom-gate bias provides an electrical means of adjusting the current response while balancing response amplitude and quiescent conduction. For subsequent force calibration, repeatability testing, and wearable measurements, *V*_BG_ = 0 V was selected as a suitable operating condition.

Similar piezoelectric–TFT architectures reported previously have used the electrical output of PVDF or PVDF-TrFE elements as the input to a transistor gate, thereby translating mechanically generated electrical signals into changes in drain current [15,16]. The result in Figure 2d extends this electromechanical-to-electronic conversion to the dual-gate configuration and, importantly, shows that the magnitude of the resulting current response can be adjusted electrically through the applied gate condition. This provides an additional electronic degree of freedom beyond the direct voltage response of the PVDF element itself [17,18].

**Figure 3:**
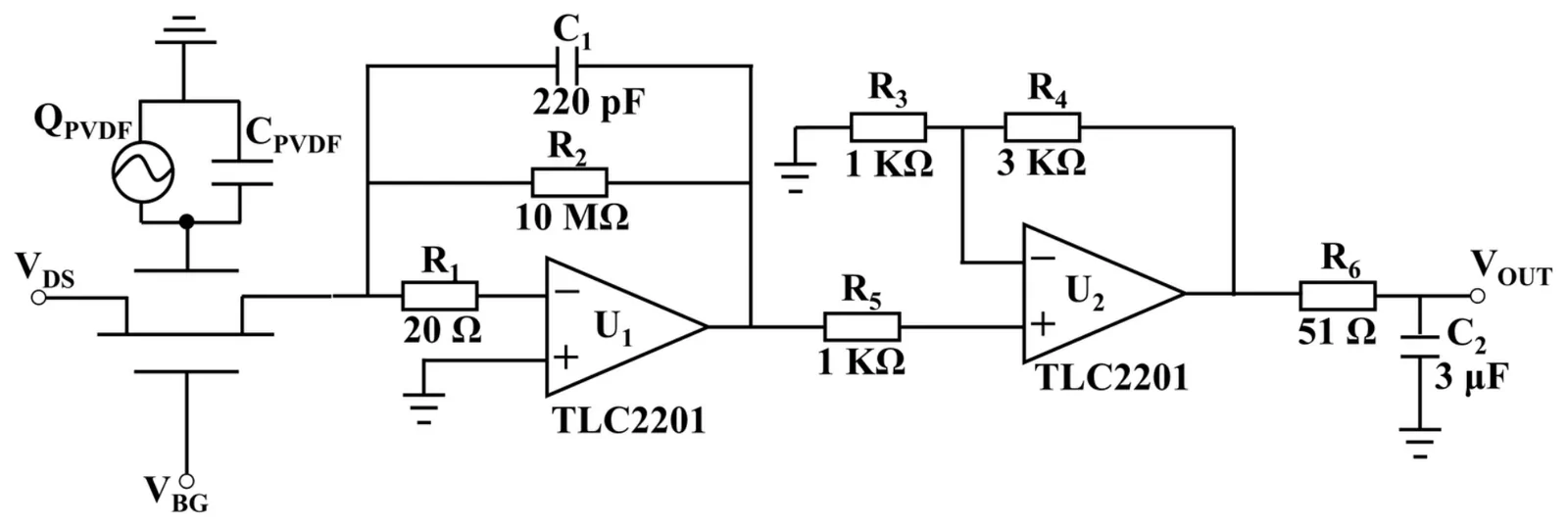
Schematic of the analog readout circuit for signal conditioning and voltage acquisition.

**Figure 4:**
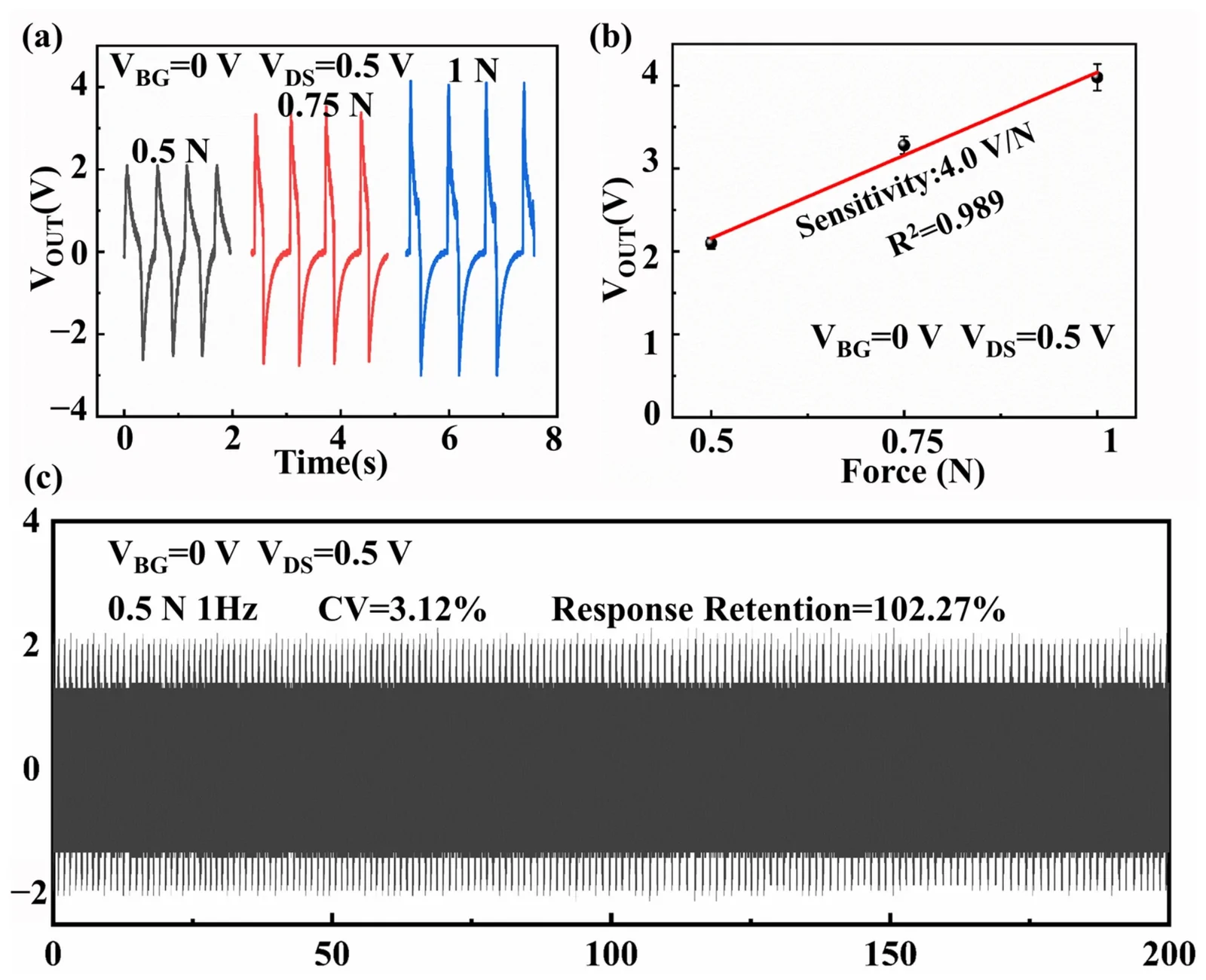
(**a**) Output-voltage responses under 0.5, 0.75, and 1 N loading. (**b**) Force-to-voltage calibration based on the mean positive peak voltages from 20 loading events at each force level. Error bars represent the sample standard deviation, and the solid line indicates the linear fit to the three means. (**c**) Short-term repeatability under nominal 0.5 N cyclic loading for 200 s.

The analog readout circuit shown in Figure 3 converts the electrical response of the PVDF-TFT sensing section into a conditioned output voltage. The first stage, *U*_1_, performs current-to-voltage conversion using the R_2_-C_1_ feedback network, which provides a nominal low-frequency transimpedance of 10 MΩ and a calculated −3 dB cutoff frequency of 72.3 Hz. The second stage employs a non-inverting voltage amplifier (*U*_2_), with feedback resistors R_3_ = 1 kΩ and R_4_ = 3 kΩ, providing a closed-loop voltage gain of 4 V/V. The amplified signal subsequently passes through an R_6_-C_2_ low-pass filter with a calculated cutoff frequency of approximately 1.04 kHz. These stages jointly determine the amplitude- and frequency-dependent characteristics of the recorded signal. In particular, the first-stage feedback network attenuates signal components above its cutoff frequency, influencing the temporal profile of the conditioned voltage. The standalone PVDF voltage in Figure 2c, the transistor current changes in Figure 2d, and the final output voltage in Figure 4 represent complementary electrical measurements at different stages of the sensing architecture. Accordingly, the measured *V*_OUT_ reflects the combined response of the piezoelectric element, transistor interface, and analog readout circuit. The same operating biases and readout configuration were maintained throughout force calibration, short-term repeatability testing, and wearable measurements, providing a consistent basis for evaluating the system-level voltage response.

Therefore, the force-to-voltage calibration was based on the conditioned output *V*_OUT_, rather than the standalone PVDF voltage or the transistor current response. The sensitivity characterizes the integrated sensing and readout configuration under the specified operating conditions. This distinction between the intrinsic transducer response and the interface-dependent electrical output is relevant to the interpretation of piezoelectric readout circuits and transistor-integrated sensing systems [19,20].

The final output of the sensing system exhibits a clear dependence on applied force. Representative *V*_OUT_ traces obtained at 0.5, 0.75, and 1 N are shown in Figure 4a. All three loading conditions generate reproducible bipolar waveforms, while their amplitudes in-crease progressively with force. Analysis of 20 consecutive loading response events at each force level yielded mean positive peak voltages of 2.099 ± 0.067 V at 0.5 N, 3.279 ± 0.105 V at 0.75 N, and 4.099 ± 0.161 V at 1 N, indicating limited event-to-event variation under the same loading condition. The similarity of the waveform profile across the three loading conditions, together with the systematic amplitude increase, provides a direct basis for quantitative calibration of the complete sensing system. The calibration curve in Figure 4b shows an approximately proportional increase in *V*_OUT_ over the investigated range. Linear regression of the three mean positive peak voltages yields a system-level force-to-voltage sensitivity of 4.0 V/N, with R^2^ = 0.989. The calibration results exhibit an approximately linear increasing trend across the investigated force levels of 0.5, 0.75, and 1 N. This relationship characterizes the response of the integrated sensing platform under the fixed operating conditions of *V*_DS_ = 0.5 V and *V*_BG_ = 0 V.

Short-term repeatability was evaluated under repeated 0.5 N loading at a nominal frequency of 1 Hz for 200 s (Figure 4c). The voltage response remained continuous throughout the measurement, with a coefficient of variation of 3.12% for the extracted positive peak-voltage sequence. The mean positive peak voltages of the first and last 10 response events were 2.112 and 2.160 V, respectively, corresponding to a final-to-initial response ratio of 102.27%. This represents a 2.27% increase in the mean positive peak voltage between the initial and final event groups. The small change in mean response, together with the low dispersion of the repeated peak voltages, indicates consistent short-term electrical behavior under the investigated loading condition.

The calibrated force-dependent voltage response and short-term repeatability provide the basis for the subsequent wearable measurements. Controlled loading establishes that different mechanical input levels can be represented quantitatively by the electrical output, while the 200 s test shows that repeated deformation produces a consistent response sequence. These two characteristics provide the experimental foundation for interpreting the neck-mounted measurements as mechanically generated signals acquired by the same sensing platform.

**Figure 5:**
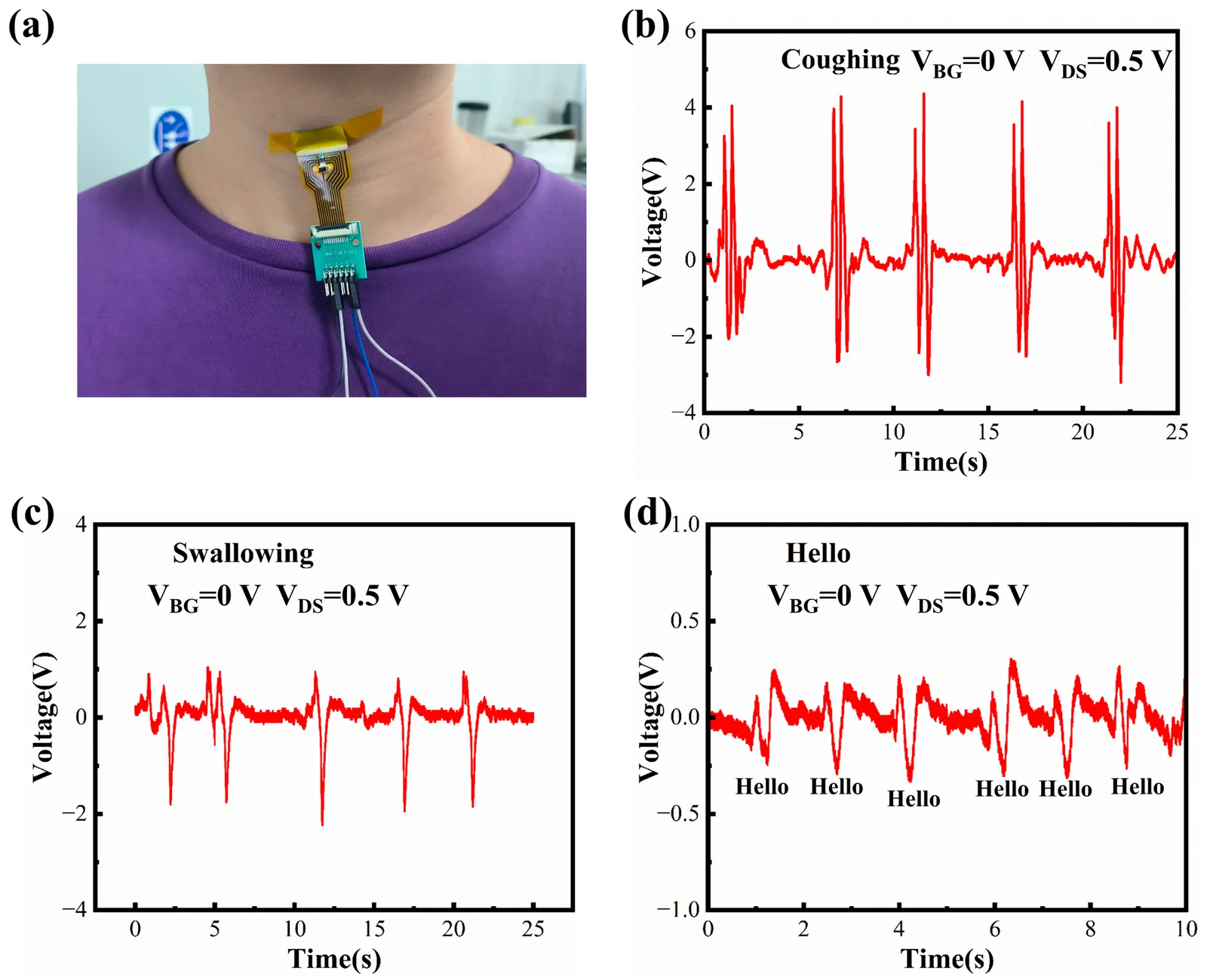
(**a**) Sensor placement on the anterior neck. Voltage responses during (**b**) five coughing events, (**c**) five swallowing events, and (**d**) six utterances of “hello” from the same participant.

The device was attached to the anterior neck near the laryngeal region for wearable mechanical sensing, as shown in Figure 5a. The complete recorded sequences of five coughing events, five swallowing events, and six utterances of “hello” are presented in Figure 5b–d, respectively. The three activities exhibit distinguishable time-domain voltage responses, with differences in signal amplitude, temporal extent, and waveform morphology. These characteristics reflect the different dynamic mechanical motions occurring at the neck surface during coughing, swallowing, and phonation.

Coughing produces the most prominent transient responses among the three activities examined. As shown in Figure 5b, repeated coughing events generate pronounced bipolar voltage transients, characterized by relatively large positive peaks accompanied by negative excursions. These responses are generally concentrated within short time intervals. Coughing involves rapid pressure release, airflow acceleration, and motion of the laryngeal and tracheal tissues, all of which contribute to fast deformation and vibration at the neck surface. The observed bipolar waveforms are consistent with this impulsive biomechanical character. Similar neck-mounted sensing studies have reported strong transient or broadband mechanical responses during coughing.

Swallowing produces broader multiphasic responses. As shown in Figure 5c, the recorded events exhibit successive voltage fluctuations, including positive and negative excursions followed by signal recovery. Swallowing is mechanically more sequential than coughing because coordinated motion of the hyoid, laryngeal structures, and surrounding musculature produces deformation of the anterior neck over an extended temporal sequence [21,22]. Previous piezoelectric throat-sensing studies have directly related neck-surface signals to hyoid and thyroid cartilage motion and have further shown that swallowing involves mechanical components associated with laryngeal displacement, muscular activity, and local vibration. The multiphasic waveforms observed here are consistent with this mechanically complex process.

Pronunciation of “hello” produces a representative signal pattern. As shown in Figure 5d, repeated utterances generate localized voltage responses with amplitudes generally lower than those observed during coughing and swallowing. Phonation originates from periodic vocal-fold vibration that propagates through the laryngeal framework and surrounding soft tissues to the neck surface [23,24]. The relatively small voltage fluctuations are consistent with the repetitive mechanical vibrations associated with vocalization rather than the larger transient displacements observed during coughing. Previous neck-mounted vibration sensors have similarly reported speech-related mechanical responses and characteristic periodic components during phonation [2,23].

The three throat activities exhibit distinguishable time-domain voltage responses in terms of amplitude, temporal extent, and waveform morphology (Figure 5b–d). Quantitative analysis of all recorded events yielded mean peak-to-peak voltages of 6.860 ± 0.497 V for coughing (mean ± SD, n = 5), 2.748 ± 0.171 V for swallowing (n = 5), and 0.522 ± 0.047 V for “hello” (n = 6). The mean voltage amplitude during coughing was approximately 2.50 times that during swallowing and 13.14 times that during pronunciation of “hello”, while swallowing produced an average amplitude approximately 5.26 times that of “hello”. The corresponding coefficients of variation were 7.25%, 6.23%, and 9.01%, respectively. These values describe the event-to-event amplitude variation across the complete recorded sequences under the single-participant measurement protocol. The differences in voltage amplitude and temporal morphology provide complementary descriptors of the mechanical responses associated with the three throat activities, supporting the feasibility of the integrated platform for wearable throat-motion monitoring. The measurements provide a quantitative description of the different throat-motion responses observed in the single-participant wearable experiment, but have not yet demonstrated the device’s versatility.

Recent throat-sensing studies have shown that such temporal differences are often accompanied by distinct spectral and time–frequency characteristics: coughing tends to contain strong transient broadband components, speech exhibits periodic and harmonic features, and swallowing involves mechanical responses distributed across different timescales [2,21]. In the present measurements, the observed differences in time-domain waveform morphology indicate that the neck-mounted PVDF platform can capture representative responses associated with these throat activities. Nevertheless, contact electrification at the skin encapsulation–adhesive interfaces, cable motion, and variations in local contact pressure may also contribute to the recorded signals. Although PI encapsulation and lead fixation were employed, these potential contributions were not independently isolated. Therefore, the results demonstrate the feasibility of wearable throat-motion sensing, while further controlled experiments are needed to distinguish piezoelectric responses from motion-related artifacts and evaluate the reliability of the recorded waveform characteristics.

## 4. Conclusions

This work presents a hybrid piezoelectric sensing architecture that integrates a pre-poled PVDF film, an ITZO dual-gate thin-film transistor, and an analog readout circuit. The integration establishes a successive signal conversion pathway in which mechanical deformation is converted into a piezoelectric potential, coupled to the transistor channel through the top gate, and subsequently transformed into a conditioned voltage output. The dual-gate configuration provides complementary functions for piezoelectric signal coupling and transistor operating point control, enabling electrical adjustment of the channel response to dynamic mechanical inputs. The integrated system achieves a force-to-voltage sensitivity of 4.0 V/N over the investigated force range of 0.5–1 N. The system also exhibits consistent electrical behavior under repeated mechanical loading, with a coefficient of variation of 3.12% during the 200 s repeatability test. In wearable measurements, the sensor captures distinct voltage patterns associated with coughing, swallowing, and phonation. The differences in signal amplitude and temporal morphology illustrate the capability of the integrated architecture to resolve mechanical variations at the anterior neck. These findings highlight the potential of combining piezoelectric transduction with transistor-based signal modulation and analog conditioning for wearable biomechanical sensing. The present work establishes a device-level approach for integrating flexible piezoelectric materials with oxide thin-film transistor technology. Further development will focus on improving the mechanical integration of the sensing element, evaluating signal reproducibility across multiple participants, and investigating the influence of sensor placement and motion-related interference. Such efforts will support the development of more reliable wearable systems for continuous throat-motion monitoring.

## Data Availability

The data presented in this study are available from the corresponding author upon reasonable request.

## Abbreviations

The following abbreviations are used in this manuscript:

PVDF	Polyvinylidene fluoride
SD	Standard deviation
SEM	Scanning electron microscopy
ITZO	Indium tin zinc oxide
TFT	Thin-film transistor
DGTFT	Dual-gate thin-film transistor
PI	Polyimide
ITO	Indium tin oxide
XRD	X-ray diffraction
AFM	Atomic force microscopy
CV	Coefficient of variation
